# The Effect of Sleep Impairment, as Assessed by the CASIS Questionnaire, in Patients with Chronic Obstructive Pulmonary Disease on Disease Severity and Physical and Mental Health: A Cross-Sectional Study in Primary Care

**DOI:** 10.3390/biomedicines12081644

**Published:** 2024-07-24

**Authors:** Izolde Bouloukaki, Antonios Christodoulakis, Katerina Margetaki, Ioanna Tsiligianni

**Affiliations:** 1 Department of Social Medicine, School of Medicine, University of Crete, Voutes-Stavrakia, 71003 Heraklion, Greece; christodoulakisa@uoc.com (A.C.); katmargetaki@hotmail.com (K.M.); i.tsiligianni@uoc.gr (I.T.); 2 Department of Nursing, School of Health Sciences, Hellenic Mediterranean University, 71410 Heraklion, Greece

**Keywords:** COPD, sleep impairment, primary care

## Abstract

The aim of our study was to assess the prevalence of sleep impairment among primary care patients with COPD and explore its impact on disease severity and overall health status. This cross-sectional study included 251 participants > 40 years old from the prospective COCARE COPD study. Data on sociodemographic characteristics, medical history, disease-specific quality of life [COPD Assessment Test (CAT)], COPD severity [Global Initiative for Chronic Obstructive Lung Disease (GOLD) 2023 categorization based on CAT score and exacerbations], fatigue [Fatigue Severity Scale (FSS)], psychological parameters [Patient Health Questionnaire-9 (PHQ-9) and General Anxiety Disorder-7 (GAD-7)], and sleep complaints [COPD and Asthma Sleep Impact Scale (CASIS) and Epworth Sleepiness Scale (ESS)] were collected. Multiple logistic regression analysis was conducted to test for associations of sleep impairment with COPD and overall health status, adjusting for confounders. Sleep impairment, indicated by a CASIS score ≥ 30, increased the risk of worse COPD health status (CAT ≥ 10 OR: 9.7, 95% CI: 5–19, *p* < 0.001), COPD severity (GOLD B OR: 8.9, 95% CI: 4.5–17.6, *p* < 0.001 and GOLD E OR: 17.4, 95% CI: 5.1–59.4, *p* < 0.001), excessive daytime sleepiness (ESS > 10, OR: 3.2, 95% CI: 1.3–8.1, *p* = 0.012), depressive symptoms (PHQ-9 ≥ 10, OR: 6.4, 95% CI: 2.1–19.1, *p* = 0.001), anxiety symptoms (GAD-7 ≥ 10, OR: 3.9, 95% CI: 1.6–9.2, *p* = 0.002), and fatigue (FSS ≥ 36, OR: 5.3, 95% CI: 2.8–9.8, *p* < 0.001). In conclusion, our findings suggest that sleep impairment, based on the CASIS questionnaire, is associated with worse physical and mental health in patients with COPD. Therefore, through consistent evaluation of sleep and targeted management strategies, healthcare providers could improve the quality of life for these patients.

## 1. Introduction

Chronic Obstructive Pulmonary Disease (COPD) is a common disease that has a significant impact on mortality, morbidity, and healthcare expenditures [1]. Globally, the prevalence of COPD is around 10.3% [2], whereas in Greece, it is estimated to affect approximately 8.4% to 10.6% of the population [3,4]. Patients with COPD frequently experience a range of complex physical symptoms that extend beyond respiratory dysfunction. These symptoms include coughing, breathlessness, and increased sputum production, as well as persistent fatigue, unintentional weight loss, and sleep disturbances [5,6,7,8], which pose significant challenges to patients’ physical and mental health, affecting their performance in everyday activities and social interactions [9].

Even though the prevalence and the negative impact of sleep impairment on overall well-being and functioning are well documented [10], patients with COPD usually do not report it or physicians do not adequately screen and address sleep quality and night-time symptoms [11,12,13,14]. More specifically, sleep-related symptoms, such as insomnia, persistently feeling unrefreshed upon awakening in the presence of a normal sleep duration, and daytime fatigue are experienced by over 60% of patients diagnosed with COPD [6,15]. Moreover, studies that utilize objective sleep measurements such as polysomnography have suggested that patients with different severities of COPD experience a decrease in sleep efficiency and a shorter duration of REM sleep [10,16]. Nevertheless, the underlying mechanisms are not well understood, but there is evidence suggesting a reciprocal link between poor sleep and the severity of COPD [17]. For example, symptoms like wheezing, sputum production, comorbidities, psychological distress, advanced age, and the use of inhaled or systemic corticosteroids, along with nocturnal hypoventilation and ventilation/perfusion abnormalities, can all lead to a deterioration in sleep quality [6,18,19]. Interestingly, it is common for patients diagnosed with COPD to also have obstructive sleep apnea (OSA), and this co-existence can further deteriorate the already impaired sleep quality of these individuals [2,20]. On the other hand, previous studies have suggested that sleep disturbances in patients with COPD could have unfavorable outcomes, including an increased likelihood of COPD exacerbations [21], neurocognitive dysfunction [17], and impaired functional exercise capacity [22].

Notably, to accurately detect and quantify sleep disturbances in COPD, it is essential to employ well-designed questionnaires. The most commonly used questionnaire to assess sleep quality in both clinical settings and research is the Pittsburgh Sleep Quality Index (PSQI). This questionnaire has been applied to patients with various medical diseases, including COPD [21,22,23,24,25,26,27,28,29,30,31,32,33,34,35,36,37]. The prevalence of poor sleep quality, as determined by the PSQI score, can range from 27.2% [35] to 85.3% [36] in patients with COPD. While this questionnaire is commonly employed in patients with COPD, conflicting results have been reported regarding its association with patient-reported outcome measures (PROMs), including physical health, depressive and anxiety symptoms, and quality of life [21,22,23,24,25,26,27,28,29,30,31,32,33,34,35,36,37].

Unlike the PSQI, the recently introduced COPD and Asthma Sleep Impact Scale (CASIS) questionnaire was designed to identify changes in sleep quality that are specifically linked to respiratory problems in these patients [38]. This tool appears to be both sensitive and specific for detecting changes in sleep quality caused by COPD, making it user-friendly for healthcare professionals in clinical practice [24,39,40,41,42,43,44,45,46,47,48,49,50,51,52]. The CASIS also has the potential to identify individuals with recurrent sleep complaints, thus serving as a valuable tool for assessing sleep impairment and its impact on outcomes associated with COPD [38,40]. In support of this, studies have indicated a correlation between CASIS score and adverse effects on COPD health status [24,38,39,40,42,43,46,47,48], mental health [24,39,46], and quality of life [47].

However, there is a lack of comprehensive studies available in the literature that adequately explore the connection between sleep quality, as assessed by the CASIS questionnaire, and self-reported health in individuals with COPD using PROMs specifically within primary care settings [40] and in Greece [40,43]. Therefore, the aim of our study was to assess the prevalence of sleep impairment based on CASIS questionnaire among primary care patients with COPD in Greece and explore its impact on COPD severity, sleepiness, insomnia symptoms, fatigue, depressive, and anxiety symptoms. 

## 2. Materials and Methods

### 2.1. Design and Sample

This cross-sectional study included patients with COPD who were invited from six primary care centers in Crete, Greece. In order to be considered for inclusion, patients had to fulfill the following requirements: (a) being at least 40 years old and having physician-diagnosed COPD confirmed through spirometry, (b) being educated beyond elementary school, and (c) providing written informed consent. Patients with severe neurological or mental disorders, pregnancy, recent exacerbation of COPD, limited comprehension of the Greek language, or a lack of willingness to participate were excluded.

### 2.2. Procedure

Information about this study’s objectives was provided to patients with COPD during their visits. After giving their consent to participate, they submitted written consent and proceeded to anonymously fill out the questionnaires. To minimize the impact of social desirability bias, participants were given instructions to place their study materials inside an opaque container positioned outside the office. 

### 2.3. Data Collection

Participants were subjected to a comprehensive evaluation that examined various parameters, including age, gender, BMI, exercise routines, tobacco and alcohol consumption, educational background, and comorbidities. Information about a patient’s self-reported health status was assessed using various PROMs (Supplementary Table S1). The COPD Assessment Test (CAT) was utilized to evaluate the quality of life specifically related to COPD. Fatigue was evaluated by utilizing the Fatigue Severity Scale, while psychological aspects were assessed through the Patient Health Questionnaire-9 and General Anxiety Disorder-7 questionnaire. The COPD and Asthma Sleep Impact Scale (CASIS), Athens Insomnia Scale, and Epworth Sleepiness Scale were used to assess subjective sleep quality and sleep-related complaints. The duration of the assessment for each participant was approximately 20–30 min.

### 2.4. Study Tools and Outcomes

#### 2.4.1. COPD Assessment Test (CAT) Questionnaire

The CAT is a simple to use questionnaire that evaluates the self-reported effects of COPD on overall health. It has been also validated in Greek [53]. There are eight items in the CAT, including cough, phlegm, chest tightness, breathlessness, limited activities, confidence in leaving home, sleeplessness, and energy. Patients rate these symptoms on a scale of 0 to 5 [54]. A higher value on the score, ranging from 0 to 40, corresponds to a poorer health status. Poor health status is determined by using a cutoff point of 10 or greater. 

#### 2.4.2. Fatigue Severity Scale (FSS)

The Fatigue Severity Scale (FSS) is administered by presenting individuals with nine items that pertain to the severity, frequency, and impact of fatigue on various aspects of daily life, such as physical functioning, exercise and work, and family or social life. The participants are then asked to rate their agreement with each item using a Likert scale ranging from 1 to 5, with 1 indicating strong disagreement and 5 indicating strong agreement. The FSS score is obtained by calculating the average of the scores assigned to each item, with higher scores indicating more severe fatigue. Scores below 36 are considered normal, while a score of 36 or above indicates a significant negative impact of fatigue on daily activities, with a maximum score of 63 [55]. The FSS has been translated and culturally adapted for use in Greek [56].

#### 2.4.3. Patient Health Questionnaire (PHQ-9)

The Patient Health Questionnaire-9 (PHQ-9) is a self-reporting tool comprising nine items. These items align with the criteria utilized in diagnosing depressive disorders, as outlined in The Diagnostic and Statistical Manual of Mental Disorders (DSM), specifically the 4th edition (DSM-IV) [57]. Additionally, the PHQ-9 is theoretically consistent with the 5th edition of the DSM (DSM-5) [42]. The PHQ-9 has a score range of 0 to 27, with higher scores indicating more severe depressive symptoms. The cutoffs of 5, 10, 15, and 20 correspond to mild, moderate, moderately severe, and severe depressive disorders, respectively. In this study, a cutoff point of 10 or higher was used to determine the presence of depressive symptoms. Furthermore, the PHQ-9 has been validated in Greek [58].

#### 2.4.4. General Anxiety Disorder (GAD-7)

The GAD-7 is a commonly used questionnaire that evaluates seven symptoms associated with generalized anxiety disorder, as outlined in the DSM-IV [59]. The total score on this instrument ranges from 0 to 21, with higher values indicating a greater level of disturbance. Scores of 5, 10, 15, or higher correspond to mild, moderate, or severe impairment, respectively. In our study, a threshold of 10 or above was utilized as a marker for the presence of GAD, indicating the existence of moderate-to-severe anxiety symptoms. The Greek version of the GAD-7 [60] was employed in the present research.

#### 2.4.5. COPD and Asthma Sleep Impact Scale (CASIS)

In this study, a Greek version of the CASIS 7-item questionnaire [40] was employed. The CASIS-7 questionnaire comprises 5 items that measure the impact of breathing difficulties/COPD on falling asleep or remaining awake during the day in the previous week. Additionally, it assesses the impact of breathing problems/COPD on 2 more items that pertain to sleep quality in the same time period. A Likert scale is employed to measure the occurrence of symptoms, with scores ranging from 1 (never) to 5 (very often). The total sum score is calculated by adding up the scores of the 7 items, which can range from 7 (representing the best outcome) to 35 (representing the worst outcome). The CASIS-7 sum score is converted into a total scale score ranging from 0 to 100 using a linear transformation equation: (CASIS-7 sum score-7) × [100/(35 − 7)]. Higher scores indicate more severe sleep impairment [38]. Based on the work of Ierodiakonou et al., sleep impairment was defined as a CASIS-7 score of 30 or higher [40].

#### 2.4.6. Athens Insomnia Scale (AIS)

The AIS is a standardized assessment tool developed in Greece for evaluating sleep difficulty according to the International Classification of Diseases-10 (ICD-10) guidelines. As a self-assessment psychometric instrument, it comprises eight items and generates a total score ranging from 0 to 24. A score of 6 or higher suggests a potential diagnosis of insomnia [61].

#### 2.4.7. Epworth Sleepiness Scale (ESS)

The Epworth Sleepiness Scale (ESS) is currently the most commonly utilized subjective measurement tool for evaluating daytime drowsiness in clinical settings [62]. It ranges from 0 to 24, and a score below 10 is indicative of normal range. In our study, the Greek version of the ESS was employed [63].

### 2.5. Statistical Analysis

The mean values for continuous variables with a normal distribution were reported alongside their standard deviation (SD), while for variables without a normal distribution, the median was provided along with the 25th and 75th percentiles. Categorical variables were represented by the absolute numerical value and the corresponding percentage. To analyze differences between groups, a two-tailed *t*-test for independent samples was employed for continuous variables following a normal distribution, while the Mann–Whitney U-test was used for variables not following a normal distribution. Additionally, the Chi-square test was applied for categorical variables. Correlations between continuous scales were determined using Pearson’s correlation coefficient.

In order to assess the associations between the CASIS-7 and the studied PROMs, we employed linear regression for the continuous CASIS scales and logistic regression for the dichotomized CASIS scales. Each PROM was then fitted into a separate model. In each model, we also included factors that were associated (*p* < 0.05) with both the CASIS and PROMs. Thus, all models were adjusted for age, gender, level of education, exercise, and BMI. We further adjusted for OSA risk by using a sensitivity analysis. Multicollinearity among the predictors was assessed using collinearity statistics to ensure that the collinearity between the predictor variables was within the acceptable range, as indicated by the tolerance value variance inflation factor (a VIF < 3 for each model). The results were deemed significant if the *p*-values were less than 0.05. Data were analyzed using Stata software (version 13).

## 3. Results

### 3.1. Patient Characteristics

A total of 251 patients with COPD were included in this study. The average age of the patients was 68 ± 9 years. Among the participants, 69% were male, 45% were obese (BMI ≥ 30 kg/m^2^), and 76% had a spouse. A significant portion of the participants had received a low level of education (49%) and a slightly smaller percentage had a middle education (36%), while 15% had a high level of education. In terms of smoking habits, 48% of the participants were actively smoking during the survey, whereas 44% had quit smoking. At least one chronic disease was present in 90% of the patients. The sociodemographic characteristics and health statuses of the 251 participants are provided in Table 1.

Table 2 displays the distribution of patients into the Global Initiative for Chronic Obstructive Lung Disease (GOLD) 2023 categories based on CAT scores and exacerbations [2]. The majority of participants (51%) were classified into GOLD group B, whereas group E accounted for less than 15%, according to the GOLD 2023 classification. In the 12-month period prior to this study, the majority (86%) of patients did not suffer from COPD exacerbations or had just one, whereas 14.3% experienced two or more exacerbations and 5.6% required hospitalization. 

Regarding sleep quality, the mean (SD) values of the CASIS-7 scale score was 37.6 (16.5). The main complaint expressed by the majority of patients was waking up feeling unrested, followed by experiencing a poor night’s sleep. Interestingly, the CASIS score appeared to differ based on educational level, physical activity status, and the severity of COPD (Table 3).

### 3.2. Differences in Clinical Characteristics of Patients with COPD with Good and Low Sleep Quality

The application of a CASIS-7 ≥ 30 cutoff to define low sleep quality led to the identification of 38.2% of patients with good sleep quality and 61.8% of patients with low sleep quality. The clinical variables of the two groups are summarized in Table 1 and Table 2. No significant differences were found in the anthropometrics and comorbidities between the groups. However, patients with lower sleep quality had lower educational levels and had worse COPD health status based on the CAT score and GOLD classifications.

### 3.3. Correlation of Sleep Quality with PROMs

Regarding PROMs, it is important to highlight that a substantial proportion of patients displayed elevated levels of fatigue (FSS) and insomnia symptoms, as assessed using the AIS. Furthermore, patients with low sleep quality exhibited the most severe functional impairments, with statistical significance observed across all the questionnaires (Table 4). It is evident that individuals who lacked adequate sleep quality experienced heightened levels of fatigue, depression, anxiety, symptoms of insomnia, and sleepiness compared to those with good sleep quality. 

After adjusting for age, gender, level of education, exercise, and BMI, it was observed that not only the total score of the CASIS-7 but also CASIS ≥ 30 displayed an inverse relationship with the CAT, FSS, PHQ-9, GAD-7, and AIS scores (Table 5). Moreover, the presence of low sleep quality was still found to be independently associated with COPD symptoms (CAT score ≥ 10) (OR = 9.7, 95% CI 5–19; *p* < 0.001), fatigue (FSS ≥ 36) (OR = 5.3, 95% CI 2.8–9.8; *p* < 0.001), EDS (ESS ≥ 11) (OR = 3.2, 95% CI 1.3–8.1; *p* < 0.001), depressive symptoms (PHQ-9 score ≥10) (OR = 6.4 95% CI 2.1–19.1; *p* < 0.001), anxiety symptoms (GAD-7 ≥ 10) (OR = 3.9, 95% CI 1.6–9.2; *p* < 0.001), and insomnia symptoms (AIS ≥ 6) (OR = 56.7, 95% CI 17.9–180; *p* < 0.001). Further adjustments for OSA diagnosis resulted in similar results.

## 4. Discussion

The objective of this study was to examine the sleep quality based on CASIS questionnaire and its association with different PROMs in primary care settings among patients with COPD. Our findings suggest that a considerable number of patients with COPD experienced poor sleep quality. Moreover, poor sleep quality was positively associated with adverse health outcomes in these patients. Such outcomes included worsened health status and increased sleepiness, fatigue, depression, anxiety, and symptoms of insomnia. Notably, these associations remained significant regardless of the participants’ age, sex, BMI, education level, and exercise status.

Our population had an average CASIS questionnaire score of 37.6, which is in line with the majority of previous studies on sleep quality in COPD populations that employed the same questionnaire [38,39,40,41,43,47,48,50,52]. Lower scores were reported in three studies [24,42,49] and higher scores were reported in one study [44]; however, these studies had relatively small sample sizes. It is also important to note that CASIS scores did not vary by gender, age, obesity, marital status, smoking history, or number of comorbidities. 

Another major finding of this study was the high prevalence of poor sleep quality, as indicated by 61.8% of participants scoring above the threshold of 30 on the CASIS questionnaire. Furthermore, the main complaint expressed by a majority of patients was waking up feeling unrested, followed by experiencing a poor night’s sleep. The significant percentage of individuals experiencing poor sleep quality is noteworthy, particularly when considering previous studies that have highlighted the substantial impact of sleep quality on disease-specific quality of life in patients with COPD [22,24,35,38,39,40,43,47,48]. The health status of our population in relation to COPD was determined by a mean CAT score of 12.3, suggesting a medium impact of COPD on overall health. Additionally, individuals with poor sleep quality had a 9.7 times higher likelihood of experiencing worsened health status linked to COPD. This was also evident from their elevated CAT scores and a higher proportion of people falling into GOLD classification groups B and E. These findings align with previous research that showed a positive correlation between higher CAT scores (indicating worse health status) in patients with COPD and higher scores on sleep quality questionnaires (indicating poor sleep quality), specifically the PSQI [24,29], and CASIS questionnaires [24,40,43]. Other studies have also supported these findings, showing a positive association between inadequate sleep quality and an increased frequency of exacerbations [21,28,29,38], as well as the severity of dyspnea [39,46,47], frequent sputum production [35], and increased CAT scores [34,40,42,43]. Given the considerable effect of sleep quality on the health of patients with COPD, it is reasonable to anticipate an increase in medication usage and healthcare resource utilization among those with poor sleep quality. The aforementioned could have affected both social and economic aspects, potentially leading to missed social events and work and a decrease in overall quality of life.

The interest in evaluating the quality of life in patients with COPD as a primary endpoint has seen significant growth in recent years. However, in Greece, there is limited research on the quality of life of patients with COPD in non-hospital environments [64,65,66]. To the best of our knowledge, this study is the first that assesses quality of life in patients with COPD using different PROMs and their correlations with sleep quality through the use of a specific questionnaire designed for COPD. PROMs, which are reports directly provided by patients on their perceived health status, play a crucial role in assessing disease severity and its impact on the health conditions of patients with COPD [67] Moreover, the data were collected from a representative sample of COPD patients from six primary care centers in Crete, Greece. Conversely, the utilization of validated PROMs allowed for a standardized approach for evaluating the effect of the disease on patients’ well-being. Furthermore, the utilization of fatigue, mental health, and sleep health questionnaires ensured a thorough evaluation of most aspects related to the health status of patients with COPD. Based on our findings, it appears that poor sleep quality has a noticeable influence on the health status of COPD patients, affecting aspects such as sleepiness, fatigue, and mental well-being.

Among the participants in our study, 16% experienced excessive daytime sleepiness (EDS), but the prevalence of this condition rose to 21% among patients who reported poor-quality sleep. Furthermore, patients who reported EDS had a 3.2-fold increased likelihood of experiencing low sleep quality. Unfortunately, there is a scarcity of pertinent research supporting these results. Two previous studies involving 100–120 patients with COPD demonstrated that EDS was prevalent in 8% [34] and 34.8% [37] of the COPD population. However, no correlation was found between EDS and sleep quality, as measured by the PSQI [22,34]. Our study also found a significant number of participants (75.5%) experiencing symptoms of insomnia, which aligns with previous research findings, showing a prevalence as high as twice of that of the general population [6,23,27,28,68,69]. Various factors such as nocturnal dyspnea, decreased physical activity and outdoor time, diminished exposure to bright light, and medication for COPD could be implicated in this high prevalence of insomnia in COPD populations [70]. The presence of low sleep quality was strongly associated with insomnia symptoms, with a significant odds ratio of 56.7, even after accounting for potential confounders. Therefore, these findings suggest the need for further research to determine the role of sleep quality and insomnia in daytime sleepiness among these patients.

Another finding of our study was that the health statuses of these patients were further affected by fatigue, as reported by 66% of our participants. Fatigue is one of the most prevalent and challenging symptoms that requires effective management [71,72]. Patients with COPD experience limitations in performing routine activities due to fatigue, and despite its significance as a debilitating symptom, it is often overlooked and not properly evaluated in these patients [73]. Despite the insufficient evidence available in the literature, there has been a growing recognition of sleep as a substantial factor in the development of fatigue among individuals with COPD [72]. Our study suggests that patients with poor sleep quality were 5.3 times more prone to having this symptom. Therefore, by obtaining a more thorough understanding of the factors correlated with COPD-related fatigue, we could uncover new approaches to address this and ultimately offer relief to a greater number of these patients.

Mental disorders, especially depression, have been identified as significant psychological factors directly associated with fatigue [74]. It is widely acknowledged that individuals with COPD often experience mental health issues, such as anxiety (ranging from 10% to 50%) and depression (30%), greatly affecting their overall well-being [75,76,77]. The results of our study showed a relatively lower, relative to previous studies, prevalence of depressive (17.2%) and anxiety symptoms (21.2%), suggesting that mental disorders may have been overlooked in these patients. However, patients who reported lower sleep quality demonstrated significantly elevated levels of depression (25.2%) and anxiety (28.9%) symptoms, whereas those with good sleep quality had much lower rates (4.3% and 8.6%, respectively). Furthermore, having poor sleep quality was linked to a 6.4 times greater likelihood of experiencing depressive symptoms and a 3.9 times greater likelihood of experiencing anxiety symptoms. Previous studies [24,26,32] have also described these associations, providing further evidence that poor sleep quality is associated with symptoms of anxiety and depression. It is worth noting that anxiety and depression have been found to have a bidirectional relationship with sleep dysregulation [78]. Importantly, sleep disturbances seem to elevate the risk of developing anxiety and depression two-fold, and the co-occurrence of depression and sleep disturbance amplifies the probability of hospitalization in COPD by a factor of five [79]. Consequently, effectively diagnosing and treating sleep disorders can help to prevent the development or worsening of coexisting anxiety or depression, and vice versa. One approach to more effectively manage patients with COPD in the community could be to routinely ask them about their sleep habits and any related concerns during initial or follow-up assessments. Confirmatory questions could be asked, regardless of the possibility of a negative response, such as “Did you wake up feeling refreshed?” or “Did you go to bed at a regular time?”. Additionally, the patient’s partner/caregiver could offer valuable information and insights about the symptoms experienced by the patient. Considering the significance of assessing sleep impairment in these patients, it would be highly advantageous for GPs to incorporate questionnaires, such as the CASIS, during their assessments.

There are some limitations to our study that should be underlined. First, we utilized a cross-sectional research design, which precluded us from determining causal relationships between sleep quality and PROMs. Despite this limitation, cross-sectional studies offer valuable insights into the associations between various variables and can aid in the development of future prospective investigations. Therefore, future research could utilize a longitudinal design that incorporates crucial measures, such as mortality rates, hospitalizations caused by exacerbations, and overall quality of life. Second, it is challenging to make direct comparisons with other studies that have employed different methods to assess sleep quality. Third, as the CASIS questionnaire primarily aims to identify changes in sleep quality related to respiratory symptoms of COPD, the potential indirect influence of COPD symptom severity on the independent association between sleep impairment and adverse health outcomes should be considered. Lastly, a majority of the patients in our study were categorized as A or B in the GOLD classification, whereas only a small percentage belonged to group E. This restricted the generalizability of our findings to populations with more severe COPD.

## 5. Conclusions

In conclusion, our findings suggest that sleep impairment is associated with worse health status and increased fatigue, depression, anxiety, and insomnia symptoms in patients with COPD. Therefore, by having knowledge about the factors that affect the health of individuals with COPD, healthcare professionals could provide more precise and personalized treatment. Moreover, sleep problems in patients with COPD could affect the patients’ quality of life and serve as predictors of unfavorable clinical outcomes, as suggested by various PROMs, particularly in primary care settings, where healthcare professionals have more face-to-face interactions with their patients. Consequently, regular evaluation of sleep by healthcare professionals, especially in primary care, followed by targeted management strategies, could significantly improve the quality of life for these patients.

## Figures and Tables

**Table 1:** Demographic characteristics of the participants (*n* = 251).

Characteristics	Overall	Good Sleep Quality CASIS < 30	Low Sleep Quality CASIS ≥ 30	*p*-Value
	*n* = 251 (100%)	*n* = 96 (38.2%)	*n* = 155 (61.8%)	
**Age (years)**	68.0 ± 9.0	68.1 ± 9.0	67.9 ± 9.0	0.868
Age group 40–50 years	11 (4.4)	4 (4.2)	7 (4.5)	0.987
Age group 51–64 years	80 (31.9)	31 (32.3)	49 (31.6)	
Age group ≥ 65 years	160 (63.7)	61 (63.5)	99 (63.9)	
**Gender**				
Male	172 (68.5)	68 (70.8)	104 (67.1)	0.536
Female	79 (31.5)	28 (29.2)	51 (32.9)	
**BMI (kg/m^2^)**	30.2 ± 6.4	29.5 ± 6.4	30.6 ± 6.4	0.194
**BMI ≥ 30 kg/m^2^**	113 (45.0)	40 (41.7)	73 (47.1)	0.401
**Have a spouse**				
Yes	191 (76.1)	71 (74.0)	120 (77.4)	0.532
No	60 (23.9)	25 (26.0)	35 (22.6)	
**Smoking**				
Active	120 (48.0)	44 (46.3)	76 (49.0)	0.332
Former	110 (44.0)	46 (48.4)	64 (41.3)	
Never	20 (8.0)	5 (5.3)	15 (9.7)	
Pack Years	67.8 ± 39.3	63.5 ± 35.4	70.6 ± 41.6	0.182
**Alcohol (units/week)**	0.0 (0.0, 7.0)	1.5 (0.0, 14.0)	0.0 (0.0, 7.0)	0.195
**Physical Exercise (min/week)**	0.0 (0.0, 210.0)	0.0 (0.0, 240.0)	0.0 (0.0, 200.0)	0.015
Exercise < 120 min/week	152 (61.8)	52 (54.7)	100 (66.2)	0.071
Exercise ≥ 120 min/week	94 (38.2)	43 (45.3)	51 (33.8)	
**Education level**				
Primary level	118 (48.6)	38 (40.4)	80 (53.7)	0.012
Secondary level	88 (36.2)	34 (36.2)	54 (36.2)	
Higher level	37 (15.2)	22 (23.4)	15 (10.1)	
**Co-morbidities**				
Arterial Hypertension	137 (54.8)	55 (57.3)	82 (53.2)	0.532
CVD	83 (33.1)	33 (34.4)	50 (32.3)	0.729
Diabetes type 2	76 (30.3)	32 (33.3)	44 (28.4)	0.407
Hyperlipidemia	145 (57.8)	58 (60.4)	87 (56.1)	0.504
Osteoporosis	32 (12.7)	11 (11.5)	21 (13.5)	0.629
Cancer	27 (10.8)	5 (5.2)	22 (14.2)	0.026
Depression	32 (12.7)	12 (12.5)	20 (12.9)	0.926
Anxiety disorder	14 (5.6)	5 (5.2)	9 (5.8)	0.841
Obstructive Sleep Apnea	40 (16)	17 (17.9)	22 (14.5)	0.473

Data are presented as *n* (%) for categorical variables and mean values ± SD or median (25th–75th percentile) for continuous variables; BMI: Body Mass Index; CVD: cardiovascular disease.

**Table 2:** Patient disease characteristics (*n* = 251).

Characteristics	Overall	Good Sleep Quality CASIS < 30	Low Sleep Quality CASIS ≥ 30	*p*-Value
	*n* = 251 (100%)	*n* = 96 (38.2%)	*n* = 155 (61.8%)	
**CAT score**	12.3 ± 5.9	8.5 ± 4.4	14.7 ± 5.4	<0.001
CAT score ≥ 10	164 (65.3)	33 (34.4)	131 (84.5)	<0.001
**Exacerbations in the past year, *n* (%)**				
≤1	215 (85.7)	89 (92.7)	126 (81.3)	0.012
≥2	36 (14.3)	7 (7.3)	29 (18.7)	
≥1 hospitalization	14 (5.6)	4 (4.2)	10 (6.5)	0.443
**GOLD groups *n* (%)**				
A	87 (34.7)	63 (65.6)	24 (15.5)	<0.001
B	127 (50.6)	29 (30.2)	98 (63.2)	
E	37 (14.7)	4 (4.2)	33 (21.3)	

Data are presented as mean values ± SD or median (25th–75th percentile), unless otherwise indicated. CAT: COPD Assessment Test; GOLD: Global Initiative for Chronic Obstructive Lung Disease.

**Table 3:** Distribution of the CASIS-7 according to demographic and patient characteristics (*n* = 251).

Characteristics	CASIS-7 Scale Score Mean ± SD		Characteristics	CASIS-7 Scale Score Mean ± SD	*p*-Value
**Age group**		0.640	**Education level**		
40–50	42.2 ± 17.7		Primary level	38.0 ± 15.9	0.027
51–64	37.3 ± 15.3		Secondary level	39.9 ± 18.4	
65+	37.5 ± 17.0		Higher level	31.2 ± 13.1	
**Gender**		0.075	**Comorbidities**		
Male	36.4 ± 16.0		No comorbidity	36.3 ± 13.3	0.682
Female	40.4 ± 17.3		Comorbidities ≥ 1	37.8 ± 16.8	
**BMI**		0.091	**Physical Exercise**		0.008
BMI < 30 kg/m^2^	36.1 ± 15.6		Exercise < 120 min/week	39.6 ± 17.3	
BMI ≥ 30 kg/m^2^	39.6 ± 17.3		Exercise ≥ 120 min/week	33.9 ± 14.0	
**Have a spouse**		0.928	**CAT score**		<0.001
No	37.8 ± 17.7		CAT score < 10	26.4 ± 11.0	
Yes	37.6 ± 16.1		CAT score ≥ 10	43.6 ± 15.8	
**Smoking habits**		0.166	**GOLD groups**		<0.001
Active	38.8 ± 17.5		A	26.4 ± 11.0	
Former	35.7 ± 15.2		B	41.9 ± 15.4	
Never	42.2 ± 15.9		E	49.3 ± 16.0	

**Table 4:** Questionnaires scores of the 251 patients according to CASIS.

Symptoms	Overall	Good Sleep Quality CASIS < 30	Low Sleep Quality CASIS ≥ 30	*p*-Value
	*n* = 251 (100%)	*n* = 96 (38.2%)	*n* = 155 (61.8%)	
**Daytime symptoms**				
**Fatigue**				
FSS	42.5 (27.0, 46.5)	27.0 (18.0, 36.0)	45.0 (36.0, 54.0)	<0.001
FSS ≥ 36, *n* (%)	160 (65.6)	39 (41.9)	121 (80.1)	<0.001
**Daytime sleepiness**				
ESS	3.0 (3.0, 9.0)	3.0 (2.0, 4.0)	6.0 (3.0, 9.0)	<0.001
ESS ≥ 11, *n* (%)	39 (16.1)	7 (7.7)	32 (21.2)	0.006
**Depressive symptoms**				
PHQ-9	4.0 (3.0, 8.0)	2.0 (1.0, 4.0)	6.0 (4.0, 10.0)	<0.001
PHQ-9 ≥ 10, *n* (%)	42 (17.2)	4 (4.3)	38 (25.2)	<0.001
**Anxiety symptoms**				
GAD-7	6.0 (3.0, 8.0)	4.0 (2.0, 7.0)	7.0 (4.0, 10.0)	<0.001
GAD-7 ≥ 10, *n* (%)	52 (21.2)	8 (8.6)	44 (28.9)	<0.001
**Insomnia symptoms**				
Athens Insomnia Scale Score	8.3 ± 4.1	4.8 ± 2.7	10.5 ± 3.1	<0.001
Athens Insomnia Scale Score ≥ 6, *n* (%)	182 (75.5)	39 (41.9)	143 (96.6)	<0.001

Data are presented as mean values ± SD or median (25th–75th percentile), unless otherwise indicated.

**Table 5:** Adjusted associations between sleep impairment and PROMs, estimated by multivariate linear and logistic regression models.

Symptoms	*n*	CASIS Scale Score		Low Sleep Quality CASIS ≥ 30	
		Beta (95% CI)	*p*-Value	OR (95% CI)	*p*-Value
**CAT score**	238	1.5 (1.2, 1.8)	<0.001	1.3 (1.2, 1.4)	<0.001
CAT score ≥ 10	238	16.1 (12, 20.2)	<0.001	9.7 (5, 19)	<0.001
**Gold Groups**					
B vs. A	238	15.0 (10.9, 19.2)	<0.001	8.9 (4.5, 17.6)	<0.001
E vs. A	238	21.7 (15.3, 28.0)	<0.001	17.4 (5.1, 59.4)	<0.001
**Daytime symptoms**					
**Fatigue**					
FSS (range 9–63)	232	0.7 (0.5, 0.8)	<0.001	1.1 (1.1, 1.1)	<0.001
FSS ≥ 36	232	13.4 (9.1, 17.6)	<0.001	5.3 (2.8, 9.8)	<0.001
**Daytime sleepiness**					
ESS (range 0–24)	231	1.6 (1.2, 2)	<0.001	1.2 (1.1, 1.3)	<0.0010
ESS ≥ 11	231	13 (7.3, 18.7)	<0.001	3.2 (1.3, 8.1)	0.012
**Depressive symptoms**					
PHQ-9 (range 0–27)	231	2.1 (1.7, 2.6)	<0.001	1.5 (1.3, 1.7)	<0.0010
PHQ-9 ≥ 10	231	17.4 (12.1, 22.7)	<0.001	6.4 (2.1, 19.1)	0.001
**Anxiety symptoms**					
GAD-7 (range 0–21)	232	1.1 (0.6, 1.6)	<0.001	1.2 (1.1, 1.3)	<0.0010
GAD-7 ≥ 10	232	9.9 (4.6, 15.2)	<0.001	3.9 (1.6, 9.2)	0.002
**Insomnia symptoms**					
Athens Insomnia Scale Score (range 0–24)	229	3.3 (3, 3.6)	<0.001	2.2 (1.8, 2.8)	<0.001
Athens Insomnia Scale Score ≥ 6	229	22.5 (18.5, 26.5)	<0.001	56.7 (17.9, 180)	<0.001

Effect estimates are expressed for a 1-unit increase in each of the continuous scales. All models are adjusted for participants’ age, sex, education, exercise, and BMI (BMI < 30 vs. BMI ≥ 30 kg/m^2^). VIFS for all models were <2.5.

## Data Availability

The data that support the findings of this study are available from the corresponding author upon reasonable request.

## Supplementary Materials

The following supporting information can be downloaded via this link: https://www.mdpi.com/article/10.3390/biomedicines12081644/s1, Table S1: Key features of Questionnaires used for this study.
