# Supplementary material for: The Effect of Sleep Impairment, as Assessed by the CASIS Questionnaire, in Patients with Chronic Obstructive Pulmonary Disease on Disease Severity and Physical and Mental Health: A Cross-Sectional Study in Primary Care
Source: Biomedicines. 2024 Jul 24;12(8):1644. doi: 10.3390/biomedicines12081644 (PMC11351845; doi:10.3390/biomedicines12081644)
Supplement: Supplementary file 1 [file biomedicines-12-01644-s001.zip › biomedicines-3045233-supplementary.pdf]

Supplementary Table S1 . Key features of Questionnaires used for this study.

Questionnaires	Items	Cronbach's alpha
**Copd Assessment Test (CAT) Questionnaire**	Participants are asked to evaluate their current situation by assigning a rating from 0 (never) to 5 (very often) for each item listed below	0.78
	1.Cough<br>*2. Phlegm*<br>*3. Chest tightness*<br>*4. Breathlessness*<br>*5. Limited activities*<br>*6. Confidence in leaving home*<br>*7. Sleeplessness*<br>*8. Energy*	
**Fatigue Severity Scale (FSS)**	Participants are asked to express their agreement during the past week with each item below by utilizing a Likert scale that ranges from 1 (strong disagreement) and 5 (strong agreement)	0.99
	1. My motivation is lower when I am Fatigued<br>*2. Exercise brings on my fatigue*<br>*3. I am easily fatigued*<br>*4. Fatigue interferes with my physical Functioning*<br>*5. Fatigue causes frequent problems for me*<br>*6. My fatigue prevents sustained physical Functioning*<br>*7. Fatigue interferes with carrying out certain duties and responsibilities*<br>*8. Fatigue is among my most disabling Symptoms*<br>*9. Fatigue interferes with my work, family, or social life*	
**Patient Health Questionnaire (PHQ-9)**	Participants are asked to express their agreement over the last two weeks with each item below by utilizing a scale that ranges from (not at all) to 3 (nearly every day).	0.86
	1. Little interest or pleasure in doing things<br>*2. Feeling down, depressed, or hopeless*<br>*3. Trouble falling or staying asleep, or sleeping too much*	

	4. Feeling tired or having little energy<br>*5. Poor appetite or overeating*<br>*Feeling bad about yourself or that you are a failure or have let yourself or your family down*<br>*6.Trouble concentrating on things, such as reading the newspaper or watching television*<br>*7.Moving or speaking so slowly that other people could have noticed. Or the opposite being so figety or restless that you have been moving around a lot more than usual*<br>*8.Thoughts that you would be better off dead, or of hurting yourself*<br>*An item was also added to the end of the diagnostic portion of the PHQ-9 asking patients who checked off any problems on the questionnaire: "How difficult have these problems made it for you to do your work, take care of things at home, or get along with other people?"*	
**General Anxiety Disorder (GAD-7)**	Participants are asked to express their agreement over the last two weeks with each item below by utilizing a scale that ranges from (not at all) to 3 (nearly every day).	0.93
	1. Feeling nervous, anxious, or on edge<br>*2. Not being able to stop or control worrying*<br>*3. Worrying too much about different things*<br>*4. Trouble relaxing*<br>*5. Being so restless that it is hard to sit still*<br>*6. Becoming easily annoyed or irritable*<br>*7. Feeling afraid, as if something awful might happen*	
**Copd And Asthma Sleep Impact Scale (CASIS)**	Participants are asked to express their agreement during last week with each item below by utilizing a Likert scale ranging from 1 (never) to 5 (very often).	0.72
	During the past week, how often did you:<br>*1. have a bad night's sleep?*<br>*2. have problems staying awake during the day?*<br>*3. have trouble falling asleep?*<br>*4. wake up at night with breathing problems (shortness of breath, coughing, chest tightness, etc.)?*<br>*5. wake up during the night and have trouble falling back asleep?*<br>*6. have a good night's sleep?*<br>*7. wake up feeling rested?*	

**Athens Insomnia Scale (AIS)**	Participants are asked to evaluate their current situation over the past month by assigning a rating from 0 to 5 for each item listed below	0.85
	1.Sleep induction<br>*2.Awakenings during the night*<br>*3.Final awakening*<br>*4.Total sleep duration*<br>*5.Sleep quality*<br>*6.Well-being during the day*<br>*7.Functioning capacity during the day*<br>*8.Sleepiness during the day*	
**Epworth Sleepiness Scale (ESS)**	Participants are asked to express their agreement with each item below by utilizing a scale that ranges from (no chance of dozing) to 3 (high chance of dozing).	0.75
	How likely are you to doze off or fall asleep in the following situations, in contrast to feeling just tired?<br>1.Sitting and reading<br>2.Watching TV<br>3.Sitting, inactive in a public place (e.g. a theatre or a meeting)<br>4.As a passenger in a car for an hour, without a break<br>5.Lying down to rest in the afternoon when circumstances permit<br>6.Sitting and talking to someone<br>7.Sitting quietly after a lunch without alcohol<br>8. In a car, while stopped for a few minutes in the traffic	
